# Activation of the Jasmonic Acid Plant Defence Pathway Alters the Composition of Rhizosphere Bacterial Communities

**DOI:** 10.1371/journal.pone.0056457

**Published:** 2013-02-12

**Authors:** Lilia C. Carvalhais, Paul G. Dennis, Dayakar V. Badri, Gene W. Tyson, Jorge M. Vivanco, Peer M. Schenk

**Affiliations:** 1 School of Agriculture and Food Sciences, The University of Queensland, Brisbane, Queensland, Australia; 2 Australian Centre for Ecogenomics, School of Chemistry and Molecular Biosciences, The University of Queensland, Brisbane, Queensland, Australia; 3 Advanced Water Management Centre, The University of Queensland, Brisbane, Queensland, Australia; 4 Department of Horticulture and Landscape Architecture and Center for Rhizosphere Biology, Colorado State University, Fort Collins, Colorado, United States of America; University of the West of England, United Kingdom

## Abstract

Jasmonic acid (JA) signalling plays a central role in plant defences against necrotrophic pathogens and herbivorous insects, which afflict both roots and shoots. This pathway is also activated following the interaction with beneficial microbes that may lead to induced systemic resistance. Activation of the JA signalling pathway via application of methyl jasmonate (MeJA) alters the composition of carbon containing compounds released by roots, which are implicated as key determinants of rhizosphere microbial community structure. In this study, we investigated the influence of the JA defence signalling pathway activation in *Arabidopsis thaliana* on the structure of associated rhizosphere bacterial communities using 16S rRNA gene amplicon pyrosequencing. Application of MeJA did not directly influence bulk soil microbial communities but significant changes in rhizosphere community composition were observed upon activation of the jasmonate signalling pathway. Our results suggest that JA signalling may mediate plant-bacteria interactions in the soil upon necrotrophic pathogen and herbivorous insect attacks.

## Introduction

During their lifecycle, plants are exposed to a wide-range of top-down selection pressures such as pathogen attack and herbivory. Abiotic and biotic stresses including necrotrophic pathogen infection, wounding and insect attack elicit signals that trigger a phosphorylation cascade leading to jasmonic acid (JA) biosynthesis. JA is then detected by receptors which activate a signal transduction pathway involved in the expression of JA-responsive genes, resulting in the formation of chemical and physical barriers against the pathogen or herbivore [1], but also reduced overall growth. JA signalling is effective against necrotrophic pathogens [2], but the same pathway is also activated when plants interact with beneficial microbes that lead to induced systemic resistance (ISR; [3]). *Arabidopsis thaliana* mutants that are impaired in the production of the JA precursor linolenic acid are more attacked than wild-type plants by the root chewing fungus gnat *Bradysia impatiens*. Exogenous application of JA on leaves, however, reduces the susceptibility of these mutants to attack, which implies that the JA signalling pathway is involved in the induction of below-ground plant defences [4], [5]. Activation of JA signalling by exogenous supply of methyl jasmonate (MeJA) has also been shown to increase the release of signalling compounds such as flavonoids and indoles from plant roots [6], [7], [8], [9]. Given that the quantity and composition of carbon-containing compounds released by roots is known to influence rhizosphere bacterial community structure [10], activation of the JA signalling pathway is likely to enrich for certain populations over others. Nonetheless, a DDGE-based investigation of this phenomenon did not detect any influence of JA signalling pathway activation on the diversity of rhizosphere bacterial communities [11].

The root microbiome of *Arabidopsis thaliana* was recently characterised using 16S rRNA gene amplicon pyrosequencing [12], [13]. This approach enabled detailed inventories of rhizosphere microbial communities to be collected in parallel. In this study, we used the same method to test the hypothesis that rhizosphere bacterial diversity is influenced by the activation of the JA signalling pathway in *A. thaliana*. Operational Taxonomic Unit (OTU) lists and their relative abundances derived from MeJA-treated and control plant rhizospheres were used to assess changes in composition, richness, and evenness of bacterial communities.

## Materials and Methods

### Plant growth conditions, experimental treatments and rhizosphere soil sampling

Soil was collected in March 2011 from the Michigan Extension Station, Benton Harbor, MI, USA (N42°05′34″, W86°21′19″; elevation, 192 m above sea level), from an area of fallow land where *A. thaliana* had been growing naturally for several years. A total of 60 wild-type *Arabidopsis thaliana* (Col-0) plants were grown in homogenised soil within a controlled environment chamber (Percival Scientific, Boone, IA, USA) at 24°C with a light intensity of 150 µmol m^−2^ s^−1^. At the 8–12 leaf stage, plants were treated with 0.5% (v/v) methyl jasmonate (MeJA) dissolved in ethanol as described previously [14], [15]. Control plants were mock-treated with the solvent ethanol. Following a 72 h incubation period, plants were harvested and roots with attached rhizosphere soil (that closely attached to roots) were stored in Lifeguard™ Soil Preservation Solution (MO BIO Laboratories, Carlsbad, CA) at −20°C until they were processed. Plants were cultivated in two trays, whose positions within the growth chamber were changed daily throughout the experiment. Three biological replicates were used per treatment by combining soil attached to roots collected from 10 plants per replicate. Six pots of soil were also placed in the growth chamber as part of a control experiment aimed at determining whether MeJA application had a direct influence on soil microbial diversity. Three pots were treated with MeJA and three were mock-treated with ethanol, as described above. After 72 h, two grams of soil was sampled from each pot and stored at −20°C until further processing.

### Samples DNA extraction, PCR amplification, sequencing and data processing

Total DNA was extracted from two grams of soil per sample using the PowerSoil® DNA Isolation kit (MO BIO Laboratories, Carlsbad, CA). The quality of the extracted DNA was verified on a 1% agarose gel. DNA concentrations were determined using a Qubit™ fluorometer with Quant-iT dsDNA BR Assay Kits (Invitrogen) and then normalised to 10 ng µl^−1^. Eubacterial and Archaeal 16S rRNA genes were amplified by PCR in 50 µl volumes containing 20 ng DNA, molecular biology grade water, 1× PCR Buffer minus Mg^2+^ (Invitrogen), 50 nM of each of the dNTPs (Invitrogen), 1.5 mM MgCl_2_ (Invitrogen), 0.3 mg BSA (New England Biolabs), 0.02 U Taq DNA Polymerase (Invitrogen), and 8 µM each of the primers: 803F (5′-ATTAGATACCCTGGTAGTC-3′) and 1392wR (5′-ACGGGCGGTGWGTRC-3′) modified on the 5′ end to contain the 454 FLX Titanium Lib L adapters B and A, respectively. The reverse primer contained a 5–6 base barcode sequence positioned between the primer sequence and the adapter. This primer pair amplifies preferentially archaeal and bacterial DNA and avoided amplification of host (plant) eukaryotic DNA. A unique barcode was used for each sample. Thermocycling conditions were as follows: 95°C for 3 min; then 30 cycles of 95°C for 30 s, 55°C for 45 s, 72°C for 90 s; then 72°C for 10 min. Amplifications were performed using a Veriti® 96-well thermocycler (Applied Biosystems). Amplicons were purified using a QIAquick PCR purification kit (Qiagen), quantified using a Qubit™ fluorometer with a Quant-iT dsDNA BR Assay Kit and then normalised to 25 ng µl^−1^ and pooled for 454 pyrosequencing. Sequencing was performed by the Australian Centre for Ecogenomics at the University of Queensland (Brisbane, Australia).

Sequence data were processed as described previously [16]. Briefly, sequences were quality filtered and dereplicated using the QIIME script split_libraries.py with the homopolymer filter deactivated [17] and then checked for chimeras against the GreenGenes database using UCHIME ver. 3.0.617 [18]. Homopolymer errors were corrected using Acacia [19]. Sequences were then subjected to the following procedures using QIIME scripts with the default settings: 1) sequences were clustered at 97% similarity, 2) a representative sequence was randomly selected within each cluster, 3) GreenGenes taxonomy was assigned to the cluster representatives using BLAST, 4) tables with the abundance of different operational taxonomic units (OTUs) and their taxonomic assignments in each sample were generated. The number of reads was then normalised to 1,550 per sample by re-sampling the OTU table to allow comparisons of diversity without the bias of uneven sampling effort. The mean number of OTUs (observed richness) and Simpson's Diversity Index values [20] corresponding to 1,550 sequences per sample were calculated using QIIME. The observed richness and Simpson's Diversity Index values reflect the richness (number of OTUs) and equitability (evenness of population abundances within a sample) of microbial communities, respectively. The effect of MeJA treatment on the richness and equitability of bulk soil and rhizosphere bacterial communities was investigated using GLM. The effect of MeJA treatment on the composition of bacterial communities was investigated using Redundancy Analysis (RDA) with subsequent Monte-Carlo permutation tests (999 permutations) for significant testing. RDA was performed using Hellinger transformed OTU abundances [21]. All analyses were implemented using R (version 2.12.0).

## Results and Discussion

**Figure 1:**
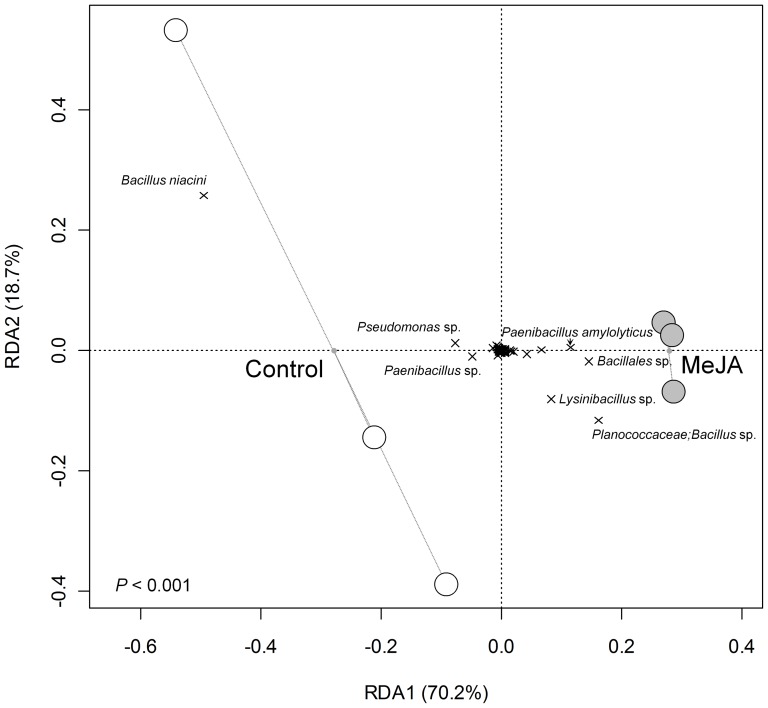
Redundancy analysis summarising variation in the composition of rhizosphere bacterial communities that can be attributed to the activation of the JA signalling pathway. White circles represent control samples and grey circles represent MeJA treated samples. The dotted lines connect each of the control and MeJA-treated samples to their respective group centroid, which is labelled and marked as a small grey dot along the primary axis. OTUs are represented by black crosses, and the taxonomic affiliation of the most discriminating of these populations is labelled.

**Figure 2:**
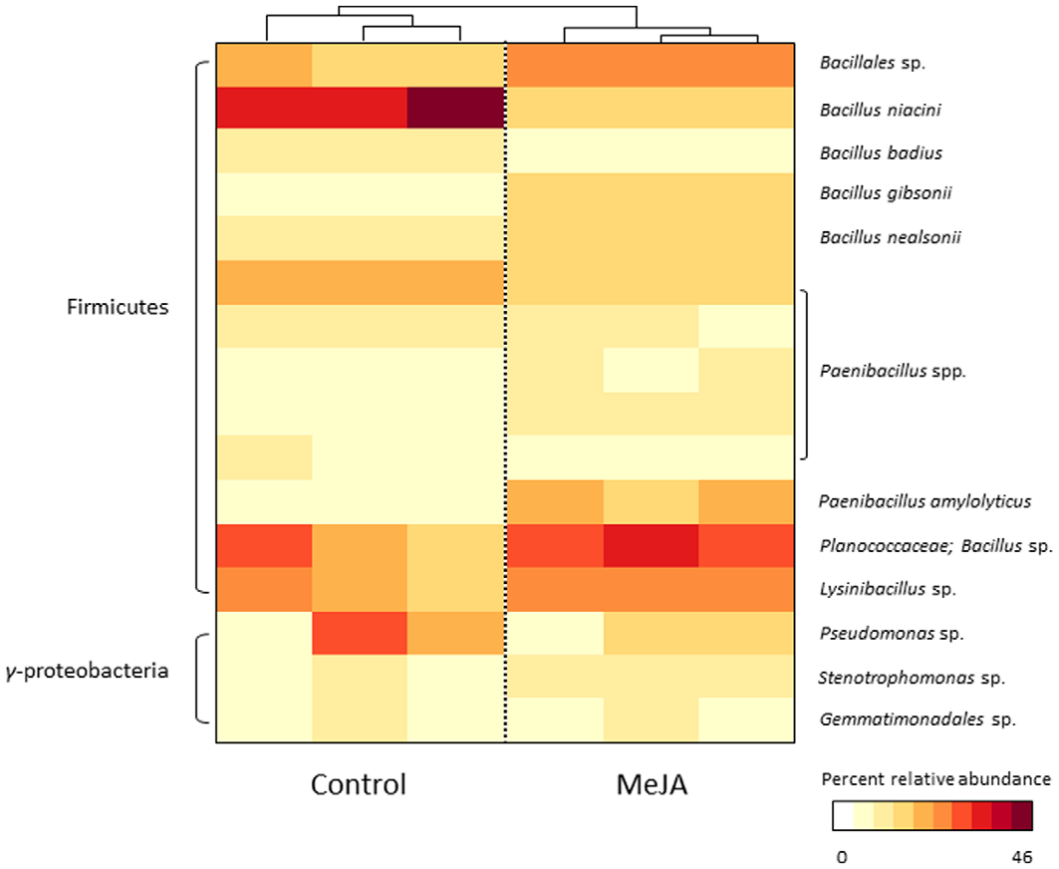
Heatmap summarising the percent relative abundances of bacteria that were present at more than 1% in any of the control or MeJA-treated samples. The relative similarity of each sample in terms of bacterial community composition as determined by complete linkage cluster analysis of OTU abundances is represented at the top of the heatmap.

Activation of the JA signalling pathway significantly influenced the composition of rhizosphere bacterial communities (Fig. 1; *P*<0.001, Redundancy Analysis (RDA)), but the richness and evenness of communities were unaffected (Table 1; P>0.05, GLM). MeJA treatment did not significantly influence the richness and evenness (P>0.05, GLM) or the composition (P>0.05, RDA) of bulk soil microbial communities (Fig. S1). This indicates that MeJA did not directly affect soil microorganisms and that the observed changes in rhizosphere community composition are related to the activation of the JA signalling pathway in plants. The composition of rhizosphere bacterial communities was also more variable for control plants than for MeJA-treated plants (Fig. 1). This suggests that JA-activated defence mechanisms culminate in a selective pressure on rhizosphere bacteria. Sixteen OTUs, all of which were representatives of the Firmicutes and Gammaproteobacteria, were present at more than 1% relative abundance in any of the control or MeJA-treated samples (Fig. 2). All of these bacteria are commonly detected in *Arabidopsis thaliana* rhizosphere soil [12], [13]. Interestingly, bacterial populations that were enriched upon activation of JA signalling were closely related to organisms that are reported to be involved in plant defence. OTUs that were observed at higher abundance in MeJA-treated relative to control plants included: 1) a *Bacillus* population from the Planococcaceae family, 2) a relative of the *Bacillales*, 3) a *Paenibacillus amylolyticus*-like representative and 4) a *Lysinibacillus*-related population (Figs. 1 & 2). The *Bacillus* population is a close relative of the strain MHS022, which is known to produce the antifungal volatiles acetamide and benzothiazole [22]. Furthermore, the *Bacillales* population is closely related to *Bacillus cereus* and *Bacillus thuringiensis*, which are known to produce toxins that are associated with biological control of insects (Cry, Cyt and Vip; [23]; [24]; [25]). *Paenibacillus amylolyticus* can suppress disease caused by *Fusarium oxysporum* infection in tomato [26], and a closely related species, *Paenibacillus alvey* protects *Arabidopsis* against leaf pathogens by triggering ISR [27]. The *Lysinibacillus* population was closely related to *Lysinibacillus sphaericus*, which has been reported to produce insecticidal toxins [28], [29]. Plants rely heavily on chemical defences against herbivorous insects. Constitutive and inducible expression of defence-related compounds occurs in both roots and shoots, which suggests that they are equally a target of attack [30]. Certain strains of *B. thuringiensis* are very efficient biocontrol agents against a number of leaf-eating insects [31], despite being found mostly in soils [24]. It is noteworthy that, although only 17% of insect families with herbivorous species include root-attacking gall makers, chewers, or sap suckers, they account for considerable agricultural losses [30]. Consequently, plants may have evolved mechanisms to recruit insecticide-producing rhizosphere bacteria when challenged by root-attacking herbivore insects.

**Table 1:** Richness and equitability of rhizosphere bacterial communities associated with control and MeJA treated *Arabidopsis thaliana* plants.

	Observed OTU (richness)	Simpson's Diversity Index (equitability)
Control 1	319.5	0.905
Control 2	288.0	0.892
Control 3	282.0	0.768
MeJA 1	315.9	0.914
MeJA 2	251.8	0.928
MeJA 3	259.6	0.923

Values are rarefied means based on 50 re-samplings of 1550 individual sequences per sample.

On the other hand, populations that were suppressed were related to bacteria that are associated with other mechanisms of plant growth promotion, such as the production of phytohormones, plant growth regulators and other biologically active substances, or by modulating the availability of nutrients or toxic elements. For example, an OTU most closely related to *Bacillus niacini* was strongly suppressed upon activation of the JA signalling pathway (Figs. 1 & 2). *B. niacini* is implicated to enhance plant growth by: 1) mobilising phosphorus, 2) producing indoles, NH_3_ and proteases, as well as 3) conferring resistance to heavy metals [32], [33]. OTUs related to a *Pseudomonas* sp. and a *Paenibacillus* sp. were also suppressed upon activation of the JA signalling pathway (Figs. 1 & 2). A BLAST comparison of the corresponding sequences against the GreenGenes database has shown that these OTUs are most closely related to *Pseudomonas putida* and *Pseudomonas wynnii*/*graminis*, respectively. *P. putida* strains have been reported to enhance plant growth by: 1) producing 1-aminocyclopropane-1-carboxylic acid (ACC) deaminase, siderophores and phytohormones, 2) mobilising phosphorus, and 3) fixing nitrogen [34], [35], [36], [37]. *Pseudomonas wynnii* and *Pseudomonas graminis* are also known to fix nitrogen and *Paenibacillus* spp. are often implicated as plant growth promoters [38], [39]. As plant roots and rhizosphere are colonised by soil bacteria which are attracted by rhizodeposits [40], [10], it is possible that roots manipulate the composition of microbial communities when they need to allocate resources for plant defence. The diversity of rhizosphere bacterial communities has been previously shown to differ between *Arabidopsis thaliana* salicylic acid-mediated systemic acquired resistance mutants [41]. Our results demonstrate that the diversity of rhizosphere bacterial communities is also influenced by JA signalling, which suggests that inducible plant defences may represent important mechanisms by which plants influence their associated microbial communities. Bacteria that are mostly involved in plant growth promotion may be suppressed, while bacteria that mainly act as biological control agents, such as insecticidal toxin- and antimicrobial-producing bacteria may be enriched. Possible candidates of signalling compounds modulating these interactions are secondary metabolites such as the phenol kaempferol-3-*O*-ß-d-glucopyranoside-7-*O*-α-l-rhamnoside, which has been shown to be released at higher rates when roots were subjected to MeJA treatment [6]. Future work should focus on confirming the roles of representative isolates of microbes found to be affected by plant JA signalling. As most bacteria cannot be cultured at present, metagenomics and metatranscriptomics [42] approaches represent complementary culture-independent alternatives as they would allow assessing rhizosphere microbial functions associated with the activation of the JA signalling defence pathway.

### Conclusions

Due to their sessile lifestyle, plants have developed a wide range of chemical defences against biological threats [30]. Maintenance of plant defences is costly; therefore, plants have evolved mechanisms that enable them to switch the allocation of resources to defence or growth [43], [44]. Our study indicates that activation of the JA signalling pathway alters the composition of rhizosphere microbial communities. This shift was associated with populations that are closely related to bacteria that are known to suppress plant pathogens and herbivore attacks. Although in several instances the same bacterial species/strain can have several plant beneficial attributes that are not mutually exclusive, it appears that when plants are not under attack, dominant rhizosphere bacterial populations are likely to be more directly related to plant growth. These observations indicate that plants may recruit a range of disease-suppressive microbes on an as-needed basis.

## Supporting Information

Figure S1 **Principal component analysis summarising variation in the composition of bulk soil microbial communities that were MeJA or mock (control) treated.** White circles represent control samples and grey circles represent MeJA treated samples. OTUs are represented by black crosses, and the taxonomic affiliation of the most discriminating populations is labelled.
(TIF)

## References

[1] Kazan K, Manners JM (2008) Jasmonate signaling: Toward an integrated view. Plant Physiol 146: 1459–1468. 18390489 10.1104/pp.107.115717 PMC2287326

[2] Laluk K, Mengiste T (2010) Necrotroph attacks on plants: wanton destruction or covert extortion? Arabidopsis Book 8: e0136. 22303261 10.1199/tab.0136 PMC3244965

[3] Van Wees SCM, Van der Ent S, Pieterse CMJ (2008) Plant immune responses triggered by beneficial microbes. Curr Opin Plant Biol 11: 443–448. 18585955 10.1016/j.pbi.2008.05.005

[4] McConn M, Creelman RA, Bell E, Mullet JE, Browse J (1997) Jasmonate is essential for insect defense in *Arabidopsis* . P Natl Acad Sci USA 94: 5473–5477. 10.1073/pnas.94.10.5473 PMC24703 11038546

[5] Puthoff DP, Smigocki AC (2007) Insect feeding-induced differential expression of *Beta vulgaris* root genes and their regulation by defense-associated signals. Plant Cell Rep 26: 71–84. 16858553 10.1007/s00299-006-0201-y

[6] Badri DV, Loyola-Vargas VM, Du J, Stermitz FR, Broeckling CD, Iglesias-Andreu L, et al (2008) Transcriptome analysis of *Arabidopsis* roots treated with signaling compounds: a focus on signal transduction, metabolic regulation and secretion. New Phytol 179: 209–223. 18422893 10.1111/j.1469-8137.2008.02458.x

[7] Buer CS, Imin N, Djordjevic MA (2010) Flavonoids: New roles for old molecules. J Integr Plant Biol 52: 98–111. 20074144 10.1111/j.1744-7909.2010.00905.x

[8] Faure D, Vereecke D, Leveau JHJ (2009) Molecular communication in the rhizosphere. Plant Soil 321: 279–303.

[9] Hassan S, Mathesius U (2012) The role of plant flavonoids in root-rhizosphere signalling: opportunities and challenges for improving plant-microbe interactions. J Exp Bot 63: 3429–3444. 22213816 10.1093/jxb/err430

[10] Dennis PG, Miller AJ, Hirsch PR (2010) Are root exudates more important than other sources of rhizodeposits in structuring rhizosphere bacterial communities? FEMS Microbiol Ecol 72: 313–327. 20370828 10.1111/j.1574-6941.2010.00860.x

[11] Doornbos RF, Geraats BPJ, Kuramae EE, Van Loon LC, Bakker PAHM (2011) Effects of jasmonic acid, ethylene, and salicylic acid signaling on the rhizosphere bacterial community of *Arabidopsis thaliana* . Mol Plant Microb Interact 24: 395–407. 10.1094/MPMI-05-10-0115 21171889

[12] Lundberg DS, Lebeis SL, Paredes SH, Yourstone S, Gehring J, et al (2012) Defining the core of *Arabidopsis thaliana* root microbiome. Nature 488: 86–90. 22859206 10.1038/nature11237 PMC4074413

[13] Bulgarelli D, Rott M, Schlaeppi K, Ver Loren van Themaat E, Ahmadinejad N, et al (2012) Revealing structure and assembly cues for *Arabidopsis* root-inhabiting bacterial microbiota. Nature 488: 91–95. 22859207 10.1038/nature11336

[14] Schenk PM, Kazan K, Wilson I, Anderson JP, Richmond T, Somerville SC, et al (2000) Coordinated plant defense responses in Arabidopsis revealed by microarray analysis. Proc Natl Acad Sci U S A 97: 11655–11660. 11027363 10.1073/pnas.97.21.11655 PMC17256

[15] Campbell EJ, Schenk PM, Kazan K, Penninckx IAMA, Anderson JP, Maclean DJ, et al (2003) Pathogen-responsive expression of a putative ATP-binding cassette transporter gene conferring resistance to the diterpenoid sclareol is regulated by multiple defense signaling pathways in Arabidopsis. Plant Physiol 133: 1272–1284. 14526118 10.1104/pp.103.024182 PMC281622

[16] Dennis PG, Guo K, Imelfort M, Jensen P, Tyson GW, Rabaey K (2013) Spatial uniformity of microbial diversity in a continuous bioelectrochemical system. Bioresource Technol In press. 10.1016/j.biortech.2012.11.098 23313735

[17] Caporaso JG, Kuczynski J, Stombaugh J, Bittinger K, Bushman F D, et al (2010) QIIME allows analysis of high-throughput community sequencing data. Nature Methods 7: 335–336. 20383131 10.1038/nmeth.f.303 PMC3156573

[18] Edgar RC, Haas BJ, Clemente JC, Quince C, Knight R (2011) UCHIME improved sensitivity and speed of chimera detection. Bioinformatics 27: 2194–2200. 21700674 10.1093/bioinformatics/btr381 PMC3150044

[19] Bragg L, Stone G, Imelfort M, Hugenholtz P, Tyson GW (2012) Fast, accurate error-correction of amplicon pyrosequences using Acacia. Nat Methods 9: 425–426. 22543370 10.1038/nmeth.1990

[20] Simpson EH (1949) Measurement of species diversity. Nature 163: 688.

[21] Legendre P, Gallagher E (2001) Ecologically meaningful transformations for ordination of species data. Oecologia 129: 271–280. 28547606 10.1007/s004420100716

[22] Zou CS, Mo MH, Gu YQ, Zhou JP, Zhang KQ (2007) Possible contributions of volatile-producing bacteria to soil fungistasis. Soil Biol Biochem 39: 2371–2379.

[23] Bravo A, Likitvivatanavong S, Gill SS, Soberon M (2011) *Bacillus thuringiensis*: A story of a successful bioinsecticide. Insect Biochem Molec 41: 423–431. 10.1016/j.ibmb.2011.02.006 PMC3689885 21376122

[24] Raymond B, Johnston PR, Nielsen-LeRoux C, Lereclus D, Crickmore N (2010) *Bacillus thuringiensis*: an impotent pathogen? Trends Microbiol 18: 189–194. 20338765 10.1016/j.tim.2010.02.006

[25] Yu X, Liu T, Liang X, Tang C, Zhu J, Wang S, et al (2011) Rapid detection of vip1-type genes from *Bacillus cereus* and characterization of a novel vip binary toxin gene. FEMS Microbiol Lett 325: 30–36. 22092859 10.1111/j.1574-6968.2011.02409.x

[26] Validov S, Kamilova F, Qi S, Stephan D, Wang JJ, Makarova N, et al (2007) Selection of bacteria able to control *Fusarium oxysporum* f. sp *radicis*-lycopersici in stonewool substrate. J Appl Microbiol 102: 461–471. 17241352 10.1111/j.1365-2672.2006.03083.x

[27] Tjamos SE, Flemetakis E, Paplomatas EJ, Katinakis P (2005) Induction of resistance to *Verticillium dahliae* in *Arabidopsis thaliana* by the biocontrol agent K-165 and pathogenesis-related proteins gene expression. Mol Plant Microbe Interact 18: 555–561. 15986925 10.1094/MPMI-18-0555

[28] Berry C (2012) The bacterium, *Lysinibacillus sphaericus*, as an insect pathogen. J Invertebr Pathol 109: 1–10. 22137877 10.1016/j.jip.2011.11.008

[29] Hu X, Fan W, Han B, Liu H, Zheng D, Li Q, et al (2008) Complete genome sequence of the mosquitocidal bacterium *Bacillus sphaericus* C3-41 and comparison with those of closely related *Bacillus* species. J Bacteriol 190: 2892–2902. 18296527 10.1128/JB.01652-07 PMC2293248

[30] Rasmann S, Agrawal AA (2008) In defense of roots: A research agenda for studying plant resistance to belowground herbivory. Plant Physiol 146: 875–880. 18316643 10.1104/pp.107.112045 PMC2259042

[31] van Frankenhuyzen K (2009) Insecticidal activity of *Bacillus thuringiensis* crystal proteins. J Invertebr Pathol 101: 1–16. 19269294 10.1016/j.jip.2009.02.009

[32] Abou-Shanab RAI, van Berkum P, Angle JS (2007) Heavy metal resistance and genotypic analysis of metal resistance genes in gram-positive and gram-negative bacteria present in Ni-rich serpentine soil and in the rhizosphere of *Alyssum murale* . Chemosphere 68: 360–367. 17276484 10.1016/j.chemosphere.2006.12.051

[33] Yadav S, Kaushik R, Saxena AK, Arora DK (2011) Diversity and phylogeny of plant growth-promoting bacilli from moderately acidic soil. J Basic Microb 51: 98–106. 10.1002/jobm.201000098 21077114

[34] Naz I, Bano A (2010) Biochemical, molecular characterization and growth promoting effects of phosphate solubilizing *Pseudomonas* sp. isolated from weeds grown in salt range of Pakistan. Plant Soil 334: 199–207.

[35] Sgroy V, Cassan F, Masciarelli O, Del Papa MF, Lagares A, Luna V (2009) Isolation and characterization of endophytic plant growth-promoting (PGPB) or stress homeostasis-regulating (PSHB) bacteria associated to the halophyte *Prosopis strombulifera* . Appl Microbiol Biot 85: 371–381. 10.1007/s00253-009-2116-3 19655138

[36] Stearns JC, Woody OZ, McConkey BJ, Glick BR (2012) Effects of bacterial ACC deaminase on *Brassica napus* gene expression. Mol Plant Microbe Interact 25: 668–676. 22352713 10.1094/MPMI-08-11-0213

[37] Viruel E, Lucca ME, Sineriz F (2011) Plant growth promotion traits of phosphobacteria isolated from Puna, Argentina. Arch Microbiol 193: 489–496. 21442320 10.1007/s00203-011-0692-y

[38] Achouak W, Normand P, Heulin T (1999) Comparative phylogeny of *rrs* and *nifH* genes in the Bacillaceae. Int J Syst Bacteriol 49: 961–967. 10425751 10.1099/00207713-49-3-961

[39] Jin HJ, Tu R, Xu F, Chen SF (2011) Identification of nitrogen-fixing *Paenibacillus* from different plant rhizospheres and a novel *nifH* gene detected in the *P. stellifer* . Microbiology 80: 117–124. 21513217

[40] Lugtenberg B, Kamilova F (2009) Plant-growth-promoting rhizobacteria. Annu Rev Microbiol 63: 541–556. 19575558 10.1146/annurev.micro.62.081307.162918

[41] Hein JW, Wolfe GV, Blee KA (2008) Comparison of rhizosphere bacterial communities in *Arabidopsis thaliana* mutants for systemic acquired resistance. Microb Ecol 55: 333–343. 17619212 10.1007/s00248-007-9279-1

[42] Carvalhais LC, Dennis PG, Tyson GW, Schenk PM (2012) Application of metatranscriptomics to soil environments. J Microbiol Methods 91: 246–251. 22963791 10.1016/j.mimet.2012.08.011

[43] Bryant JP, Kuropat PJ, Cooper SM, Frisby K, Owensmith N (1989) Resource availability hypothesis of plant antiherbivore defense tested in a South-African Savanna ecosystem. Nature 340: 227–229.

[44] Coley PD, Bryant JP, Chapin FS (1985) Resource availability and plant antiherbivore defense. Science 230: 895–899. 17739203 10.1126/science.230.4728.895

