# Supplementary figures and images for: Activation of the Jasmonic Acid Plant Defence Pathway Alters the Composition of Rhizosphere Bacterial Communities
Source: PLoS One. 2013 Feb 12;8(2):e56457. doi: 10.1371/journal.pone.0056457 (PMC3570460; doi:10.1371/journal.pone.0056457)

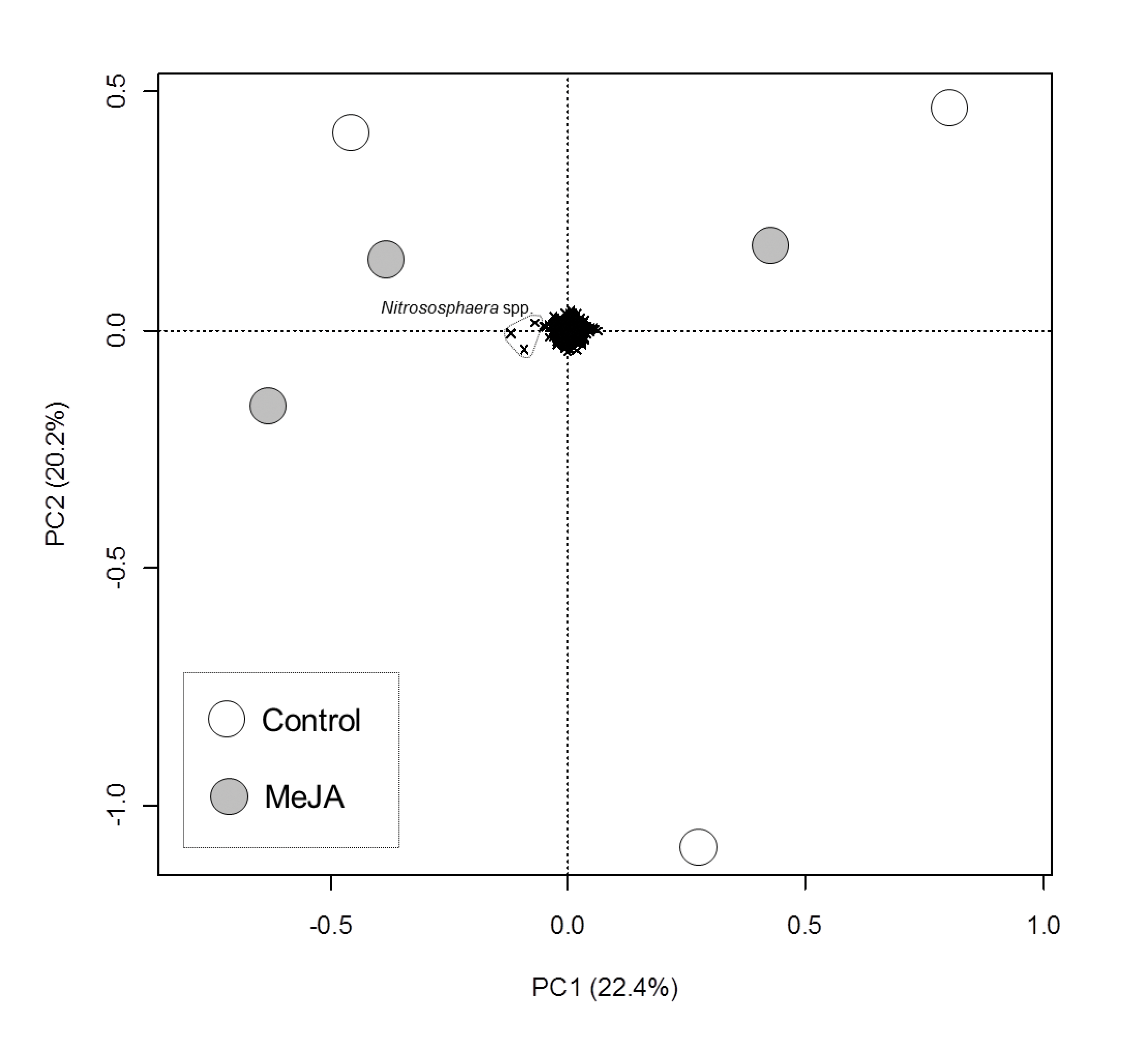

Supplement: Figure S1 — Principal component analysis summarising variation in the composition of bulk soil microbial communities that were MeJA or mock (control) treated. White circles represent control samples and grey circles represent MeJA treated samples. OTUs are represented by black crosses, and the taxonomic affiliation of the most discriminating populations is labelled. (TIF) [file pone.0056457.s001.tif]
